# Antibacterial Mechanism of Chitosan–Gentamicin and Its Effect on the Intestinal Flora of *Litopenaeus vannamei* Infected with *Vibrio parahaemolyticus*

**DOI:** 10.3390/md20110702

**Published:** 2022-11-09

**Authors:** Lefan Li, Fengyan Liang, Chengpeng Li, Tingting Hou, Dong-an Xu

**Affiliations:** 1 School of Chemistry and Environment Science, Guangdong Ocean University, Zhanjiang 524088, China; 2 Life Science &Technology School, LingNan Normal University, Zhanjiang 524048, China; 3 Mangrove Institute, LingNan Normal University, Zhanjiang 524048, China

**Keywords:** chitosan, *Vibrio parahaemolyticus*, antimicrobial mechanism, intestine microbe

## Abstract

To explore the application of chitosan–gentamicin conjugate (CS-GT) in inhibiting *Vibrio parahaemolyticus* (*V. parahaemolyticus*), which is an important pathogen in aquatic animals worldwide, the antimicrobial activity of CS-GT and the effects of a CS-GT dose on the intestine histopathology and intestinal flora of *V. parahaemolyticus*-infected shrimps were explored. The results showed that CS-GT possessed broad-spectrum antibacterial activity, with minimum inhibitory concentration (MIC), minimum bactericidal concentration (MBC) and half inhibitory concentration (IC_50_) of 20.00 ± 0.01, 75.00 ± 0.02 and 18.72 ± 3.17 μg/mL for *V. parahaemolyticus*, respectively. Further scanning electron microscope and cell membrane damage analyses displayed that the electrostatic interaction of CS-GT with cell membrane strengthened after CS grafted GT, resulting in leakage of nucleic acid and electrolytes of *V. parahaemolyticus*. On the other hand, histopathology investigation indicated that high (100 mg/kg) and medium (50 mg/kg) doses of CS-GT could alleviate the injury of a shrimp’s intestine caused by *V. parahaemolyticus*. Further 16S rRNA gene sequencing analysis found high and medium dose of CS-GT could effectively inhabit *V. parahaemolyticus* invasion and reduce intestinal dysfunction. In conclusion, CS-GT possesses good antibacterial activity and could protect shrimps from pathogenic bacteria infection.

## 1. Introduction

*V. parahaemolyticus*, a halophilic gram-negative bacterium, is a common pathogen in aquatic animals such as shrimps, crayfishes, and crabs [1,2]. It is also an important pathogen of human microbial food poisoning in coastal areas including China [3,4]. Generally, high or excessive doses of antimicrobials have been used to prevent the infection of *V. parahaemolyticus* for *Litopenaeus vannamei*. The long-term and improper use of antimicrobials might result in bacteria resistance, which poses a huge challenge to ecosystems and human health. Fortunately, functionalized natural polysaccharides with antimicrobial activities have been reported in recent years [5].

Chitosan (CS), with good biocompatibility, biodegradability and non-toxicity, has been found to possess antimicrobial activity against gram-negative bacteria, gram-positive bacteria and fungi [6,7,8]. However, its poor antimicrobial activity and aqueous solubility greatly limited its applications in aquaculture industry [9]. It is universally acknowledged that the positively charged amino groups in chitosan can interact with the negatively charged cell membrane of bacterium, disrupting its cell membrane [10]. To increase the number of active amino groups in chitosan, Luan and colleagues grafted amino groups at C-6 position in chitosan; the results indicated that the inhibitory efficiency on four plant pathogenic fungi of the modified chitosan with amino groups grafted was 20% higher than that of the original chitosan [11].

Gentamicin (GT), with five amino groups in each gentamicin molecule, is an aminoglycoside antibiotic with broad antibacterial spectrum of action. To enhance amine content in chitosan, we synthesized gentamicin-grafted-chitosan (GT-g-CS) via imine group bonding in 2019. Our experiments discovered that the antimicrobial activity of GT-g-CS for *Escherichia coli* (*E. coli*), *Pseudomonas aeruginosa* (*P. aeruginosa*), and *Staphylococcus aureus (S. aureus*) was much stronger than that of the original CS [12]. Thus, the gentamicin graft is a feasible strategy for the antimicrobial activity improvement of CS. On the other hand, imine groups in GT-g-CS are not stable, and might hydrolyze slowly in solution [13]. Inspired by the report of Huang and co-authors [14], stable chitosan–gentamicin conjugate (CS-GT) was then synthesized via reducing the imines using sodium cyanoborohydride, in 2021. Further study displayed that CS-GT could improve the resistance of *Litopenaeus vannamei* infected with *V. parahaemolyticus* via promoting the expressions of some immune-related up-regulated genes [15]. Meanwhile, the effects of a single dose of CS-GT on intestinal microbes of *Litopenaeus vannamei* were preliminarily studied, which indicated that the abundances of *Vibrio* in the gut of *V. parahaemolyticus*-infected shrimps decreased [16]. However, the antibacterial activity of CS-GT on *V. parahaemolyticus*, as well as the detailed dose effects on the intestinal flora, has not yet been explored until now. 

The intestinal microbial community, a large and most complex ecosystem, was considered to be an indispensable accessory in the life cycle of *Litopenaeus vannamei* [17,18]. Generally, a healthy gut forms a diverse, complex and stable microbiota ecosystem, which aids the host in regulating metabolic pathways, nutrient absorption and inhibiting intestinal pathogens [19]. In contrast, imbalanced gut microbiota might give rise to a disturbance in the gut´s function, causing various diseases such as metabolic syndrome, obesity, diarrhea and so on [19,20]. In recent years, investigations on the impact of dietary components or diseases on the intestinal microbiota have received increasing attention. Particularly, high-throughput sequencing technology has led to the successful catalogue of microbiota and a deep understanding of microbiota composition changes [21].

Herein, the antimicrobial activity and mechanism of CS-GT on *V. parahaemolyticus* are studied in-depth. Histopathology and 16S rRNA high-throughput sequencing were used to explore the effects of CS-GT on intestinal tissue, intestine microbial diversity and flora structure in vivo. We anticipate that this study will deepen understanding of the function of CS-GT, and provide useful guidance for *V. parahaemolyticus* control in *Litopenaeus vannamei* culture.

## 2. Results and Discussions

### 2.1. Antibacterial Activity and Mechanism of CS-GT

#### 2.1.1. Antibacterial Activity 

The antibacterial activity of CS-GT was explored through the inhibition zone diameter, firstly. According to Table 1, GT displayed the strongest inhibitory activity, with an inhibition zone diameter of 25.14 ± 0.19 mm, 23.41 ± 0.11 mm and 23.64 ± 0.07 mm for *V. parahaemolyticus*, *Helicobacter pylori (H. pylori)* and *S. aureus*, respectively. Compared with GT, the inhibition zone diameter of CS-GT is smaller (*p* < 0.05), which were 17.28 ± 0.24 mm, 18.72 ± 0.75 mm and 20.88 ± 0.84 mm, respectively. The inhibition zone diameters of CS against *V. parahaemolyticus*, *H. pylori* and *S. aureus* were 7.35 ± 0.21, 7.15 ± 0.15 and 6.81 ± 0.46 mm, respectively, which were much smaller than that of CS-GT and GT (*p* < 0.05). The results demonstrated that CS had a weak antibacterial activity against gram-negative and positive bacteria. In addition, the inhibition zone diameters of CS-GT were much larger than that of CS (*p* < 0.05), displaying that the antibacterial activity of CS-GT was significantly improved after functionalization. It is known that the amino groups of chitosan were related to the antibacterial activity, which can interact with the negatively charged bacteria cell membranes via electrostatic attractions [9]. In our study, the positively charged amino groups in CS-GT were increased after GT graft [10]. Furthermore, the effects of the imine group reduction on the antibacterial activity of CS-GT was also explored. As displayed in Table 1, the inhibition zone diameters of CS-GT for *V. parahaemolyticus*, *H. pylori* and *S. aureus* were approaching to those of GT-g-CS with no significant difference (*p* > 0.05). Therefore, the antibacterial activity of CS-GT might not change after imine groups reduced. In addition, it can be seen from the aforementioned results that CS-GT possessed the strongest inhibitory activity against *V. parahaemolyticus*. Therefore, *V. parahaemolyticus* was selected as the model bacteria for the following in-depth research.

According to Table 2, the MIC of GT was 5.00 μg/mL, which was 4 times and 64 times lower than those of CS-GT and CS (*p* <0.01), respectively. Together, the MBC was 20.00 μg/mL, which was 3.75 times and 32 times lower than that of CS-GT and GT (*p* <0.01), respectively. The results above presented that GT has a strongest inhibitory activity against *V. parahaemolyticus*. The MIC and MBC of CS-GT were 20.00 μg/mL and 75.00 μg/mL, respectively, which were 16 times and 8.53 time lower than that of CS, indicating that the antibacterial and bactericidal activity of CS were significantly improved after GT graft (*p* < 0.01).

**Figure 1:**
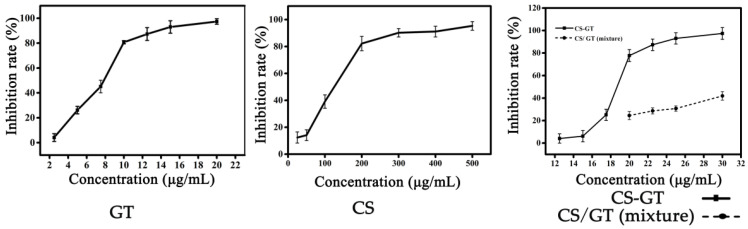
Inhibition rate of *V. parahaemolyticus* for GT, CS, CS-GT and the mixture of CS and GT (Note: Inhibition rate of the mixture of CS and GT was simulated by the real content of CS and GT in CS-GT determined by the inhibition rate curves of CS and GT. In addition, CS and GT are beyond the range of inhibition rate curve when the concentration of CS-GT is lower than 20.00 μg/mL, which was not represented here. $\bar{x}\;\pm \;\mathrm{SD},\;n\;=\;3)$.

All the inhibition rates of GT, CS-GT and CS increased with their concentration raise (Figure 1). For GT, its inhibition rate reached 97.38% at the concentration of 20.00 μg/mL, revealing the best antibacterial effect on *V. parahaemolyticus*. The inhibition rate of CS-GT was higher than 95% under a concentration of 25.00 μg/mL, which also displayed a good antibacterial effect on *V. parahaemolyticus*. However, the inhibition rate of CS was only 90% under the concentration of 300.0 μg/mL, which presented the worst antibacterial effect on *V. parahaemolyticus*. The inhibitory rates of CS-GT, GT and CS on *V. parahaemolyticus* were 77.72 ± 5.31%, 97.38 ± 3.20% and 12.36 ± 4.10% at the concentration of 20.00 μg/mL, respectively (Table S2). This indicated that the inhibition effect of CS-GT was significantly higher than that of CS (*p* < 0.01). To explore the effects of the chemical bonding on the antibacterial activity of CS-GT, the inhibition rates of CS-GT were compared to the physical mixture of CS and GT. It found that the inhibition rates of CS-GT were all much higher than that of the physical mixture of CS and GT (Figure 1). Thus, it can be deduced that GT incorporated into CS-GT can endow CS-GT with greatly improved antimicrobial activity. Furthermore, the IC_50_ of CS-GT, GT and CS are 18.72 ± 3.17, 7.89 ± 5.08 and 125.62 ± 4.35 (μg/mL), respectively, in which, CS-GT was significantly lower than that of CS (*p* < 0.01), displaying that the antibacterial efficiency of CS was significantly improved after the GT graft.

#### 2.1.2. Cell Membrane Damage

**Figure 2:**
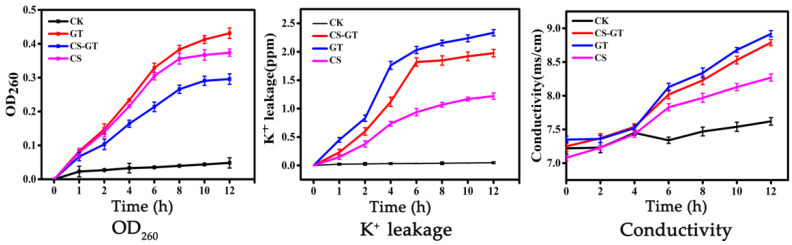
The OD_260_, K^+^ leakage and conductivity of *V. parahaemolyticus* dealing with CS, GT and CS-GT ($\bar{x\;}\;\pm \;\mathrm{SD},\;n\;=\;3$).

The integrity of the cell membrane is very important for bacteria development. Small molecules will be released first when the cell membrane is damaged, followed by the macromolecules such as RNA, DNA and other substances, which are good indicators for the integrity of the cell membrane. Among them, DNA and RNA have a strong absorption at 260 nm, which is known as “260 nm absorbing substances” [22]. So far, the detection of absorbing substances at 260 nm has been widely used to indicate the integrity of cell membrane [23]. In this study, the releases of nucleic acid (OD_260_ nm) were measured after *V. parahaemolyticus* cells were treated with CS-GT, GT and CS, which were used to evaluate the integrity of the cell membrane. As displayed in Figure 2, OD_260_ values in the control group (CK group) and the sample groups were zero at the initial time, while all the sample groups were gradually raised with the prolonged time, revealing the continual release of nucleic acid substances. GT group showed the fastest releasing, followed by CS-GT and CS groups. As can be seen from the Table S2, the OD_260_ values of CS, GT and CS-GT groups were about 5.80, 11.56 and 8.62 times than that of CK group at 12 h, and the CS-GT group was significantly lower than that of GT group and higher than that of CS group (*p* < 0.05). The results illuminated that CS-GT can cause irreparable damage to the cell membrane, resulting in the leakage of intracellular components and finally the death of cells, which was consistent with the result of a scanning electron microscope (SEM) analysis following.

Potassium ion (K^+^) plays an important role in maintaining the normal life activity of bacteria. As an innate barrier, the bacterium cell membrane is inclined to prevent potassium ions from flowing out [23,24]. A large number of potassium ions leaking to the extracellular indicates that the permeability of the cell membrane has enhanced and the cell membrane has been damaged. In this work, the permeability of the cell membranes was significantly changed after treatment with GT and CS-GT, expressed by the obvious increase of potassium ion concentration in the bacterial suspension. Almost no K^+^ leakages were detected in all groups at the initial time, while the concentration of K^+^ in all sample groups continually increased with the prolonged time interval, in which GT group showed the fastest increase trend, followed by CS-GT and CS groups (Figure 2). The concentrations of K^+^ in CS, GT and CS-GT groups increased about 24.41, 43.71 and 39.52 times at 12 h compared with the CK group (Table S2). There is no significant difference between the CS-GT and GT groups (*p* > 0.05), but the CS-GT and GT groups are significantly higher than that of the CS group (*p* < 0.05). The result demonstrated the permeability of the cell membrane, resulting in serious leakage of potassium ions, which was related to the electrostatic interaction of CS-GT with cell membrane strengthened after CS grafted GT. The result was similar to the nucleic acids leakage test.

Apart from the leakage of K^+^, other internal electrolytes such as Na^+^ and H^+^ will leak and diffuse, providing the bacterial cell membrane is destroyed by antimicrobial agents [25]. Thus, the change of electrical conductivity in the bacterial solution can also indicate the damages to the bacterial cell membrane [25,26]. As presented in Figure 2, the conductivities of *V. parahaemolyticus* suspensions in all sample groups were significantly increased with the prolonged incubation time except for the CK group. The conductivities of CS-GT, GT and CS groups increased by about 15.35%, 17.71% and 8.53% compared with the CK group after culture for 12 h (Table S2). There was no significant differences between the CS-GT and GT groups (*p* > 0.05), but both the CS-GT and GT groups were significantly higher than that of the CS group (*p* < 0.05). This result also indicated that the permeability of the cell membrane was enhanced after CS grafting GT, resulting in the leakage of intracellular electrolytes (such as K^+^, Na^+^ and H^+^), thereby the conductivity of the culture solution increased.

**Figure 3:**
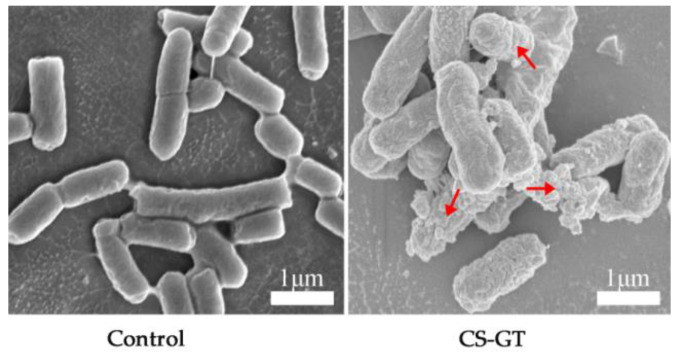
SEM images of *V. parahaemolyticus* before and after dealing with CS-GT.

Furthermore, morphological changes of bacteria were observed by SEM. The somatic cells of untreated *V. parahaemolyticus* were rod-shaped with an intact cell membrane and smooth cell outline (Figure 3). Conversely, significant morphology changes were observed for *V. parahaemolyticus* after being treated with GT-CS (Figure 3); the cell membranes were adhered and wrinkled, and the intracellular contents had leaked. The cell wall disruption may be directly related to the binding between GT-CS and proteins of the bacterial outer membrane through electrostatic interactions and hydrogen bonding [10]. The results were consistent with all the above experiments on the integrity of the cell structure. Briefly, the antibacterial activity of CS-GT on *V. parahaemolyticus* was significantly improved after CS was grafted with GT, which possessed an amount of amino groups. This is due to the positively charged ammonium groups of CS-GT increasing the electrostatic interaction with the cell membrane, resulting in the destruction of the cell membrane, the leakage of electrolytes (such as K^+^, Na^+^ and H^+^) and macromolecules (DNA and RNA) inside the cell, and finally the cell death.

### 2.2. Intestinal Histopathology

**Figure 4:**
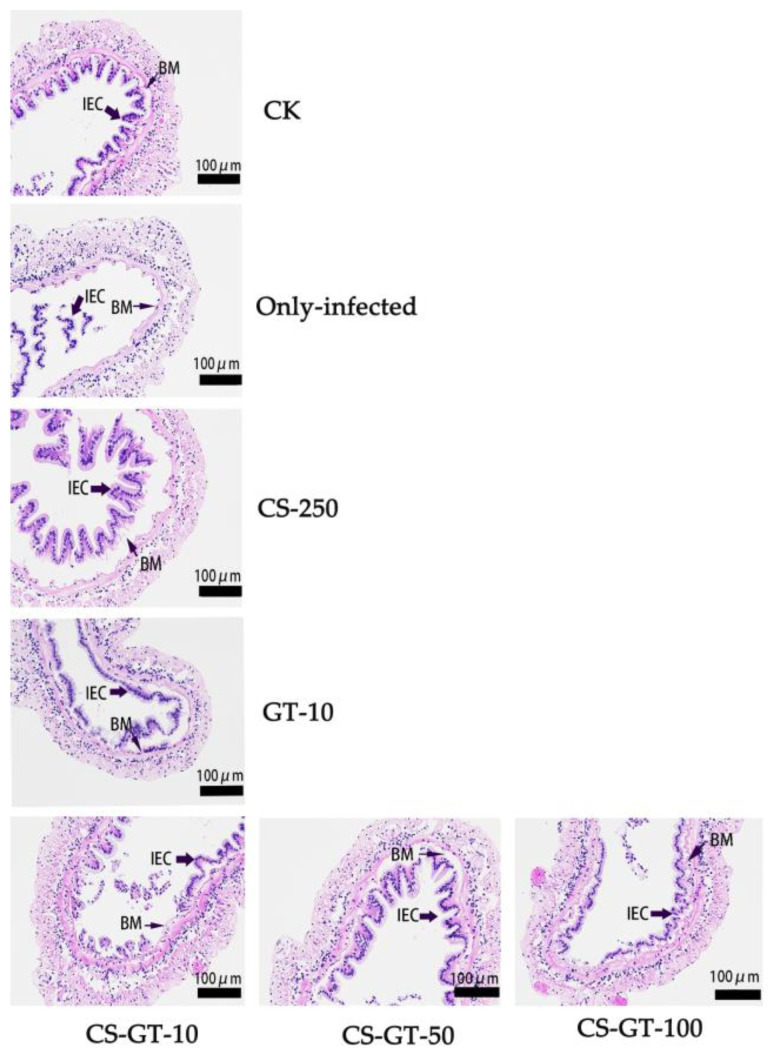
The images of cross-cutting intestine in CK group, only-infected group, CS-250 group, GT-10 group, CS-GT-10 group, CS-GT-50 group and CS-GT-100 group (*n* = 6, magnification: 200×, BM and IEC were the abbreviation of basement membrane and intestinal epithelial cells, respectively).

The integrity of intestinal morphological structures play a major role in maintenance of normal intestinal functions [27]. Changes of intestinal structure such as villus slipping and wall destruction have been related to bacterial infection. In this work, seven groups (in triplicate) were conducted, the control group (CK group, injected with saline) and the only-infected group (injected with *V. parahaemolyticus*) were fed with pure commercial feed. The other five groups, including CS-250, GT-10, CS-GT-10, CS-GT-50 and CS-GT-100 were injected with *V. parahaemolyticus* and fed with commercial feed supplemented with 250 mg/kg of CS, 10 mg/kg of GT, 10 mg/kg of CS-GT, 50 mg/kg of CS-GT and 100 mg/kg of CS-GT, respectively. The intestinal morphology was observed with hematoxylin and eosin (H&E) staining. As displayed in Figure 4, the intestine walls in the CK group were intact with neat villi and regular epithelial cells. Meanwhile, slipping of epithelial cells and villi, as well as serious damage to the intestine walls, were observed in the only-infected group, which indicated that the intestinal integrity of *Litopenaeus vannamei* was damaged after being infected with *V. parahaemolyticus*. After supplementing with 250 mg/kg of CS and 10 mg/kg of CS-GT, the intestine walls of *Litopenaeus vannamei* infected with *V. parahaemolyticus* were also severely damaged, with epithelial cells separated from the basement membrane and the villi shed in CS-250 and CS-GT-10 groups. The results indicated that 250 mg/kg of CS and 10 mg/kg of CS-GT cannot prevent intestinal structure damage caused by *V. parahaemolyticus* infection. However, the intestinal structure damage of *Litopenaeus vannamei* infected with *V. parahaemolyticus* was alleviated after GT-10, CS-GT-50 and CS-GT-100 treatment. The results showed that GT and CS-GT with medium and high dose could prevent intestinal structure damage caused by *V. parahaemolyticus* infection, to some extent. Notably, the intestinal structure integrity was improved with the increased dose of CS-GT, and the integrity of intestinal structure in the CS-GT-100 group was close to the CK group, which indicated that treatment with a high dose of CS-GT could help to prevent the intestinal injury of *Litopenaeus vannamei* caused by pathogen infections.

### 2.3. Intestinal Microbial Analysis

#### 2.3.1. Sequencing Data Analysis

The intestinal microbiota has gained much attention recently for its significant contributions towards shaping the intestinal structure through digestion of food, absorption of nutrients, competing and conquering of unfavorable microbes, and eventually improving the survival and health status of organisms [28]. Thus, the intestinal microbial composition and relative abundance were studied by barcoded 16S rRNA gene Illumina high-throughput sequencing in this work, Seven groups (in triplicate) were conducted, except for the CK group (not-infected) and the only-infected group, which were fed with pure commercial feed, the other five infected groups were fed with commercial feed supplemented with CS (250 mg/kg), GT (10 mg/kg) and CS-GT (10 mg/kg, 50 mg/kg and 100 mg/kg), respectively. After sequencing, a total of 21 (*n* = 3) intestine samples were collected from seven groups and 1,649,042 high-quality readings were obtained after data quality filtering. As shown in Figure S1, the dilution curve and species accumulation box plot gradually became flat, demonstrating that it could not obtain a large number of new operational taxonomic units (OTUs) if continued to increase the depth of sequencing. The results above displayed that the current sequencing data were sufficient to reflect the species diversity contained in the samples and supported the reliability of subsequent analysis. The Good’s coverage index in all samples was above 0.99 (Table 3), indicating that the OTUs obtained from each library effectively represented most of the microorganisms in the intestine. 

A Venn diagram was constructed to identify the common and different OTUs in different groups. The numbers of OTUs in the CK group, only-infected group, CS-GT-10 group, CS-GT-50 group, CS-GT-100 group, GT-10 group and CS-250 group were 1441, 489, 632, 619, 594, 547 and 985, respectively, and 407 OTUs were shared by all seven groups (Figure S2). Among them, OTUs of the five sample groups and the only-infected group were lower than that of CK group, and the OTUs decreased as the dose of CS-GT increased. According to the grade abundance curve (Figure S2), the species richness and uniformity of the microbial flora in the five sample groups and the only-infected group were reduced compared to the CK group. The GT-10 group, only-infected group, CS-GT-50 group and CS-GT-100 group are close to the same, and the flora richness and uniformity were reduced with the increased dose of CS-GT. 

#### 2.3.2. Microbial Diversity

The alpha diversity indices of microbial flora were displayed in Table 3. Among them, Chao1, abundance-based coverage estimator (ACE), Shannon and Observed-species indices in the only-infected, GT-10, CS-GT-10, CS-GT-50 and CS-GT-100 groups were significantly lower than that of the CK group (*p* < 0.05). In addition, the diversity and richness of the microbial flora was decreased with the raise in the CS-GT dosage. The results were consistent with the rank abundance curve, because shrimps infected with pathogens may cause an imbalance of intestinal microbial flora, while GT and CS-GT have a strong inhibitory effect on microorganisms, which also was adverse to the development of bacterial diversity.

#### 2.3.3. Microbial Flora Structure

**Figure 5:**
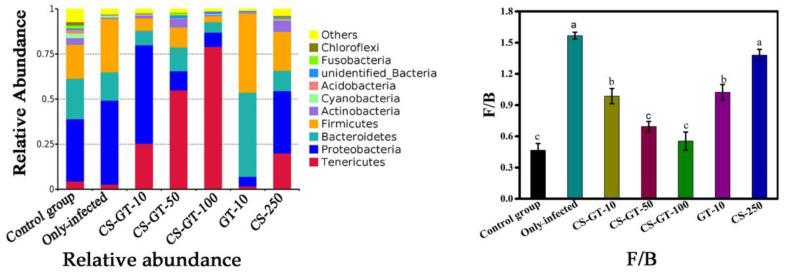
Composition and relative abundance of microbial communities at phylum level and the ratios of F/B, ($\bar{x\;}\;\pm \;\mathrm{SD},\;n\;=\;3$. Note: a, b, c indicate statistically significant variations in the same column, *p* < 0.05).

In this work, it was found that CS-GT had a great effect on the intestinal microbiota of *V. parahaemolyticus*-infected *Litopenaeus vannamei*, which was owing to the strong anti-*V. parahaemolytic* activity. Proteobacteria, including many pathogens such as *Aeromonas* and *Vibrio*, is common and abundant in the sea [29]. However, increased abundance of Proteobacteria is a signature of intestinal epithelial dysfunction [30]. As displayed in Figure 5, the abundance of Proteobacteria in the only-infected group was highest (*p* < 0.05), which was related to the *Litopenaeus vannamei* infected with *V. parahaemolytic* belonging to Proteobacteria at phylum level. The result indicated that infection with *V. parahaemolyticus* might induce gut dysbiosis of *Litopenaeus vannamei*. The abundance of Proteobacteria in the CS-GT-10 group was also significantly higher (*p* < 0.05), which indicated that a low dose of CS-GT cannot prevent the invasion and colonization of *V. parahaemolyticus* and alleviate the intestinal dysfunction. However, the abundance of Proteobacteria in the CS-GT-50, CS-GT-100 and GT-10 groups was significantly lower (*p* < 0.05), displaying that GT and CS-GT with medium and high doses can reduce intestinal dysfunction, which was consistent with the analysis of intestinal histopathology.

Bacteroidetes and Firmicutes are the main dominant bacteria in the gut microbiota at phylum level [29]. The ratio of Firmicutes to Bacteroidetes (F/B) was always considered as an important indicator of the major changes in the gut microbiota [31]. The Bacteroidetes, including Alloprevotella and Parabacteroides, has a positive effect on the gut health, while the Firmicutes, including Flaviramulus, Flavobacterium and Bacillus, has an adverse effect [31], and the increase of F/B is associated with obesity and metabolic diseases [30,31]. In our study, the F/B of CS-GT-10, CS-GT-50, CS-GT-100 and GT-10 groups was significantly lower than those of only-infected group and CS-250 group (*p* < 0.05) (Figure 5), and there was no significant difference between CS-GT-50 and CS-GT-100 groups. Particularly, the F/B of CS-GT-50 and CS-GT-100 groups were approaching to that of the CK group, which indicates that medium and high dosage of CS-GT could significantly alleviate the imbalance of intestinal microbes and metabolic diseases of shrimps infected with *V. parahaemolyticus.*

The *Vibrio* species, known as an opportunistic pathogen, is a dominant flora in the various stages of *Litopenaeus vannamei’*s developments, and it is reported to cause a lot of disease to humans and aquatic organisms [28]. The acute hepatopancreatic necrosis syndrome (AHPNS) and early mortality syndrome (EMS) caused by the *V. parahaemolyticus*, belonging to the *Vibrio* at genus level, has resulted in a large number of shrimp deaths since its occurrence [28]. The abundance of *Vibrio* was highest in the only-infected group (Figure S3); the high abundance of *Vibrio* after the *V. parahaemolyticus* challenge may be related to the invasion and colonization of *V. parahaemolyticus* in the gut of *Litopenaeus vannamei*. The abundance of *Vibrio* in the CS-250 group was higher than other sample groups (Figure S3), which indicated that 250 mg/kg of chitosan cannot effectively inhibit the invasion and colonization of *V. parahaemolyticus*. The abundances of *Vibrio* in CS-GT-10, CS-GT-50, CS-GT-100 and GT-10 groups were lower, which were approaching to the CK group. This result suggested that CS-GT, along with GT, which had a strong antibacterial activity, could inhibit the invasion and colonization of *V. parahaemolyticus*.

## 3. Materials and Methods

### 3.1. Materials

Chitosan (degree of deacetylation: 86.7%, molecular weight: 544 kDa) was bought from Qingdao Bozhihuili Biotechnology Co., Ltd. (Qingdao, China). Acetic acid, ethylene glycol, gentamicin sulfate, 4% paraformaldehyde solution, sodium periodate and sodium cyanoborohydride, provided by Shanghai Aladdin Reagent Company (Shanghai, China), were all AR grades. Liquid nitrogen was provided by Zhanjiang Oxygen Factory (Zhanjiang, China). Iuria-bertani nutrient broth (LB), tryptic soy broth (TSB), thiosulfate citrate bile salts sucrose agar culture medium (TCBS), phosphate buffer solution (PBS, pH = 7.2), tryptic soy agar (TSA) and brain heart infusion (BHI) broth were all purchased from Beijing Luqiao Technology Co., Ltd. (Beijing, China). Columbia blood agar was provided from Shandong Tuopu Biol-Engineering Co.,Ltd (Shandong, China).

*V. parahaemolyticus* and *Litopenaeus vannamei* were kindly provided by Donghai Island Marine Biology Research Base of Guangdong Ocean University (Zhanjiang, Guangdong, China). *S. aureus* (ATCC 6538) was provided by the Guangdong Microbial Culture Collection Center (Guangdong, China). *H. pylori* (ATCC 8739) was purchased from Beijing Preservation Biotechnology Co., Ltd.(Beijing, China).

### 3.2. CS-GT Synthesis

CS-GT was synthesized according to our previous report [15]. Briefly, CS was homogenously dissolved in 1.00 % (*w/v*) acetic acid, and sodium periodate solution was introduced. Then, the reaction was kept in the dark at 30 °C for 4 h, and ethylene glycol was added to terminate the reaction. Oxidized chitosan was obtained after dialysis and lyophilization. GT-g-CS was directly synthesized by mixing oxidized chitosan with gentamicin sulfate in 1.00 % (*w/v*) acetic acid solution and reacted for 4 h at 40 °C under stirring. After that, the reaction solution was cooled to 30 °C, and sodium cyanoborohydride was introduced to initiate the reduction of GT-g-CS for 2 h. After dialysis and lyophilization, CS-GT was obtained and stored for later use. The characterization of CS-GT by FTIR, XPS, ^1^HNMR, ^13^CNMR, thermal stability and elemental analysis was displayed in Figures S4–S6 and Table S4, which were described in detail in the published report [15]. According to the results of FTIR, XPS, ^1^HNMR and ^13^CNMR, GT was successfully grafted onto the CS structure, and the C=N of GT-g-CS was successfully reduced to C-N of CS-GT. In addition, the GT graft rate of CS-GT was 17.02% according to the elemental analysis. Furthermore, the stability of CS-GT was increased by 18.45% after C=N reduced to C-N. 

### 3.3. Bacterium Preparation

The activation and incubation of *V. parahaemolyticusare* were described in the literature [15]. Briefly, *V. parahaemolyticusare* was inoculated on TCBS agar medium at 37 °C for 24 h. Then, a single colony was inoculated in LB broth medium at 28 °C for 18 h. Finally, the bacterial solution was adjusted to 10^6^~10^7^ colony forming units/mL (cfu/mL) based on the absorption at 600 nm by an ultraviolet–visible spectrophotometer (756S, Lingguang Technology Co., Ltd., Shanghai, China). 

The *H. pylori* strains were inoculated in a blood agar base supplemented with 8% defibrinated horse blood (OXOID) at 37 °C for 48–72 h in a humidified microaerobic incubator (85% N_2_, 10% CO_2_, 5% O_2_). The bacterial concentration of *H. pylori* suspension, freshly prepared in saline solution was adjusted to 1 × 10^9^ cfu/mL by an ultraviolet–visible spectrophotometer for further experiments.

An *S. aureus* strain was incubated with tryptic soy broth (TSB) medium at 37 °C for 18 h. Then, the strain was diluted with TSB to obtain a concentration of 10^6^~10^7^ cfu/mL for the following use.

### 3.4. Antimicrobial Activity

#### 3.4.1. Determination of Inhibition Zones

The antimicrobial activity of samples against *V. parahaemolyticus* and *S. aureus* was determined using a standard agar diffusion method [32]. Briefly, 1.00 mL bacterial suspension (10^6^~10^7^ cfu/mL) and 10 mL TSA agar were evenly mixed, then poured into a sterile petri dish and stood horizontally for 30 min. A Whatman no. 1 sterile filter paper (diameter 6 mm) was gently put on the surface of the agar plate and stood for 30 min. A total of 10 μL of CS, GT or CS-GT solution (1.00 mg/mL) dissolved in 1.00 % (*w/v*) acetic acid was added on the filter paper, then allowed to stand for 30 min. After incubation at 37 °C for 24 h, the diameters of the inhibition zone were measured using a Vernier caliper. Each assay in this experiment was performed in triplicate.

The antibacterial activity of samples against *H. pylori* was carried out using modified agar well diffusion technique [33]. Briefly, the blood agar medium surface was inoculated with *H. pylori* suspension with a concentration of 5 × 10^4^ cfu/mL; the petri plates were left to stand for about 15 min in order to allow the culture absorption into the medium. A Whatman no. 1 sterile filter paper (diameter 6 mm) was gently put on the surface of the agar plate and stood for 30 min. A total of 10 μL of sample solution (1.00 mg/mL) dissolved in 1.00 % (*w/v*) acetic acid was added on the filter paper, which was allowed to stand for another 30 min. The plates were incubated at 37 °C for 72 h in a humidified microaerobic incubator (85% N_2_, 10% CO_2_, 5% O_2_). Finally, the mean diameter (mm) of the inhibition zone was measured. Data were recorded in three replicates for each sample.

#### 3.4.2. Determination of Minimum Inhibitory Concentration (MIC) and Minimum Bactericidal Concentration (MBC)

Samples were firstly dissolved in 1.00 % (*w/v*) acetic acid, and their MIC values were tested by being serially diluted with TSB medium in a tube [34]. An amount of 10 mL of *V. parahaemolyticus* suspension (10^6^~10^7^ cfu/mL) was transferred to the tubes. Except for the positive group containing only *V. parahaemolyticus* suspension and blank control group containing only medium, other groups had gradiently diluted samples. After incubation at 37 °C for 18 h, no visible growth of bacteria at the lowest sample concentration was determined as MIC. For the MBC test, 50 μL of bacterial suspension without visible growth above was transferred to the agar plates and incubated for 24 h at 37 °C. The lowest concentration without any visible colony on the plate was defined as MBC. Both MIC and MBC tests were repeated in triplicate and the mean values was considered as the final results. 

#### 3.4.3. Determination of Inhibition Rate and IC_50_

The inhibitory rate was determined using turbidimetric method [34]. A total of 1 mL of samples dissolved in 1.00 % (*w/v*) acetic acid with different concentrations and 1 mL of *V. parahaemolyticus* suspension (10^6^~10^7^ cfu/mL) were mixed in a sterile tube. After 24 h incubation with 120 rpm at 37 °C, the absorbance (OD_630_) was measured at 630 nm with a microplate reader (Infinite M 200 Pro, Tescan). The tubes without sample solution and bacterial suspension added were set as positive control and negative control. The tube without sample solution and bacterial suspension introduced was defined as blank control. Each assay in this experiment was replicated in triplicate. The inhibitory rate is calculated according to Formula (1):

(1) $$\mathrm{Inhibitory}\;\mathrm{rate}(\%)=\left(1-\frac{OD_{sample}-OD_{negative}}{OD_{positive}-OD_{blank}}\right)\times 100\%$$

where O*D_sample_*, O*D_positive_*, O*D_negative_* and O*D_blank_* are the absorbance of sample, positive control, negative control and blank control groups, respectively. The concentration inhibiting half of the bacterial growth is defined as IC_50_.

### 3.5. Cell Membrane Damage Assessment

#### 3.5.1. Cell membrane leakage

The leakage of nucleic acids was evaluated using the previously described method with some changes [23]. After incubation of a single colony, the *V. parahaemolyticus* suspension was centrifuged (5000 rpm at 4 °C) for 5 min and the bottom bacterial microspheres were collected. The bacterial microspheres were then washed and diluted in sterilized PBS solution with the OD_630_ value of 1.0 at 630 nm. The extracellular nucleic acid was measured at 260 nm (756S UV-visible spectrophotometer, Lingguang Technology Co., Ltd., Shanghai, China) after exposure of bacterial cells to 1 mg/mL of CS, GT or CS-GT in sterile PBS solution with 160 rpm at 37 °C. The pure sterile PBS solution was set as control group. 

The leakage of potassium ions and electrolytes of *V. parahaemolyticus* in the supernatant were determined by inductively coupled plasma spectrometer (770s, Agilent, Santa Clara, CA, USA) and conductivity meter (DDSJ-308A INESA Scientific Instrument Co., Ltd., Shanghai, China) according to the previously described methods [24,34]. Each assay was replicated in triplicate.

#### 3.5.2. Morphology Investigation

To investigate the morphology change of cell membranes, *V. parahaemolyticus* before and after CS-GT treatment was visualized using scanning electron microscopy as described previously [23]. Bacterium suspension (10^6^~10^7^ cfu/mL) was treated with CS-GT (using MIC) and incubated at 37 °C for 10 h. After centrifugation (3000 rpm at 4 °C) for 10 min, the bacterial pellet was obtained and then washed with sterile PBS buffer. The bacterial cells were fixed with an equal volume of glutaraldehyde (2.5%) at 4 °C overnight. After washing and resuspending with PBS buffer, the fixed cells were dehydrated by a series of ethanol solution (30 v%, 50 v%, 70 v%, 90 v% and 100 v%) and then freeze-dried. After gold coating, cellular morphology was observed by SEM (MIRA3, Tescan, Czech Republic) under an accelerating voltage of 20 kV.

### 3.6. Shrimp Infected with V. parahaemolyticus

Shrimps infected with *V. parahaemolyticus* performed according to the previous report [15]. *Litopenaeus vannamei* free from *V. parahaemolyticus* were randomly allotted to twenty-one tanks (300 L) with 40 shrimps in each tank, where the water temperature, pH value, salinity and dissolved oxygen content were about 30 ± 0.5 °C, 8.0 ± 0.2, 32 g/L and 6 mg/L, respectively. The rearing water was replaced with 50% seawater every day and all the shrimps were fed with commercial feed pellets at 7:00 a.m. and 5:00 p.m. twice a day.

Preliminary experiment: To obtain infected shrimps, the abdomen between the second and the third was injected with *V. parahaemolyticus* strains by intramuscular injection. Different concentrations of *V. parahaemolyticus* with 1.0 × 10^8^, 1.0 × 10^7^, 1.0 × 10^6^ and 1.0 × 10^5^ cfu/mL were used to determine the lethal concentration (LD_50_). After infection for 120 h, a concentration of 1.0 × 10^7^ cfu/mL was found to be the LD_50_. Finally, the concentration of 0.5 × 10^7^ cfu/mL was set in this work in order to ensure the sampling numbers.

Formal experiment: Seven groups (in triplicate) with different experimental diets and treatments were conducted in this study. To prepare experimental diets, samples (i.e., CS, CS-GT or GT) were wrapped on the surface of commercial feed using peanut oil. Six groups were injected with 50 μL of *V. parahaemolyticus* with a concentration of 1×10^7^ cfu/mL, while the control group (CK group) was injected with sterile PBS with the same volume. Except for the CK group and the only-infected group, which were fed with pure commercial feed, the other five infected groups were fed with commercial feed supplemented with CS (250 mg/kg), GT (10 mg/kg) and CS-GT (10 mg/kg, 50 mg/kg and 100 mg/kg) twice daily at 7:00 and 17:00 for 5 consecutive days. To keep it clean, the rearing water was replaced with 50% filtrated seawater every day.

#### 3.6.1. Intestinal Histopathology

Seven groups (in triplicate) were used in this study, which were the CK group, only-infected group, CS-250 group, GT-10 group, CS-GT-10 group, CS-GT-50 group and CS-GT-100 group, respectively. All groups were treated as above and their intestinal tissues were analyzed using the same methods in our previous report [16]. Briefly, 6 shrimps were randomly selected from each group and sacrificed after continuous administration for five days. The midgut was immediately stored in 4% paraformaldehyde and fixed for 24 h, which was then cut into cross sections (around 5 µm) after dehydration, washing and embedding. The cross-sectional specimens were stained by H&E and imaged via a light microscope (Olympus, Nikon, Tokyo, Japan).

#### 3.6.2. Intestinal Microbial Analysis

Seven groups (in triplicate) were used in this work, which were pretreated as as mentioned above. A previously described method was used to analyze the intestinal microbiology [16]. Briefly, 9 shrimps were randomly selected from each group and sacrificed after five days, the guts were immediately stored in liquid nitrogen, and the total genomic DNA was extracted. The V4 region of the bacterial 16S rDNA gene was amplified and sequenced using 515 F and 806 R as primer sets. The PCR reactions were operated as follows: initial denaturation at 98 °C for 1 min, followed by 30 cycles of denaturation at 98 °C for 10 s, annealing at 50 °C for 30 s, elongation at 72 °C for 30 s, finally keeping isothermal at 72 °C for another 5 min. After amplification, the PCR products were mixed with 1 × TAE loading buffer and detected with 2% agarose gel. Samples with a bright main strip between 400 and 450 bp were chosen, then mixed in equidensitic ratios and purified. Prior to sequencing library on Illumina HiSeq platform (Novogene, Beijing, China), the sequencing libraries were firstly generated and assessed. The sequences obtained were then analyzed using the Quantitative Insights Into Microbial Ecology software (QIIME2, Department of Chemistry and Biochemistry, University of Colorado, Boulder, CO, USA). The reads with an identity threshold of 97% were clustered into OTUs. Alpha diversities including Chao1, Shannon, ACE and Simpson were analyzed based on in-house Perl scripts. Moreover, principal coordinates analysis (PCoA) relating to the community structure similarity was performed.

### 3.7. Statistical Analysis

All data presented as $\bar{x}$ ± SD were subjected to a one-way analysis of variance (ANOVA) using Statistical Package for the Social Sciences (IBM SPSS v20.0, Inc., 2010, Chicago, IL, USA), followed by the testing of mean differences using Tukey’s multiple comparison test.

## 4. Conclusions

This work studied the antimicrobial activity and mechanism of CS-GT, and the effects of CS-GT on the intestinal structure, intestine microbial diversity and flora structure of *Litopenaeus vannamei* infected with *V. parahaemolyticus*. It found that imine group reduction of CS-GT possessed no impacts on the antibacterial activity of CS-GT, and CS-GT displayed broad-spectrum antibacterial activity toward *V. parahaemolyticus*, *H. pylori* and *S. aureus*. The MIC, MBC and IC_50_ of CS-GT are 20.00 ± 0.01, 75.00 ± 0.02 and 18.72 ± 3.17 μg/mL, respectively, presenting a good anti-*V. parahaemolytic* activity. The antibacterial mechanism of CS-GT was due to its damage to the cell membrane of *V. parahaemolyticus*, leading to leakage of nucleic acid and electrolytes. When the CS-GT dosage was 50 mg/kg or higher, CS-GT was able to reduce the invasion and colonization of *V. parahaemolyticus,* improve the intestinal integrity, and alleviate the imbalance of intestinal flora. In summary, it was speculated that CS-GT could potentially be applied for the treatment of *V. parahaemolyticus* infection in shrimp culture.

## Figures and Tables

**Table 1:** Inhibition zone diameters of bacteria dealing with CS-GT, GT, and CS ($\bar{x\;}\;\pm \;\mathrm{SD},\;n\;=\;3$).

Sample	*V. parahaemolyticus* (mm)	*H. pylori* (mm)	*S. aureus* (mm)
CS	8.60 ± 0.18 ^c^	8.14 0.06 ^c^	7.21 ± 0.05 ^c^
GT	23.64 ± 0.19 ^a^	23.01 ± 0.11 ^a^	25.14 ± 0.07 ^a^
CS-GT	20.22 ± 0.24 ^b^	17.88 ± 0.75 ^b^	18.88 ± 0.84 ^b^
GT-g-CS	20.61 ± 0.25 ^b^	18.11 ± 0.06 ^b^	19.94 ± 0.24 ^b^

Note: a,b,c indicate statistically significant variations in the same column, *p* < 0.05.

**Table 2:** MIC and MBC for *V. parahaemolyticus* ($\bar{x\;}\;\pm \;\mathrm{SD},\;n\;=\;3$).

Sample	MIC (μg/mL)	MBC (μg/mL)
CS	320.0 ± 0.00 ^a^	640.0 ± 0.00 ^a^
CS-GT	20.00 ± 0.01 ^b^	75.00 ± 0.02 ^b^
GT	5.00 ± 0.01 ^c^	20.00 ± 0.00 ^c^

Note: a, b, c indicate statistically significant variations in the same column, *p* < 0.01.

**Table 3:** Alpha diversity indices of intestinal microbiota ($\bar{x\;}\;\pm \;\mathrm{SD},\;n\;=\;3$).

Group	Observed-Species Index	Good’s Coverage Index	Richness		Diversity	
			Chao1	ACE	Shannon	Simpson
Control group	1441 ± 329 ^a^	0.9972 ± 0.0018 ^a^	1516.02 ± 354.21 ^a^	1499.89 ± 343.49 ^a^	7.91 ± 0.49 ^a^	0.8732 ± 0.0225 ^a^
Only-infected group	632 ± 100 ^b^	0.9981 ± 0.0004 ^a^	624.05 ± 105.93 ^b^	702.84 ± 99.44 ^b^	6.07 ± 0.70 ^b^	0.9537 ± 0.0178 ^a^
CS-250	1215 ± 234 ^a^	0.9975 ± 0.0004 ^a^	1267.86 ± 240.90 ^a^	1249.54 ± 238.34 ^a^	7.16 ± 0.63 ^a^	0.9172 ± 0.0124 ^a^
CS-GT-10	876 ± 236 ^b^	0.9972 ± 0.0004 ^a^	956.18 ± 155.56 ^b^	847.92 ± 331.12 ^b^	5.61 ± 1.36 ^b^	0.9250 ± 0.0695 ^a^
CS-GT-50	734 ± 198 ^b^	0.9980 ± 0.0002 ^a^	773.03 ± 203.32 ^b^	757.69 ± 184.13 ^b^	4.56 ± 2.30 ^b^	0.9404 ± 0.1016 ^a^
CS-GT-100	651 ± 263 ^b^	0.9981 ± 0.0003 ^a^	715.73 ± 256.47 ^b^	716.84 ± 247.79 ^b^	3.71 ± 0.87 ^b^	0.9209 ± 0.0615 ^a^
GT-10	611 ± 136 ^b^	0.9976 ± 0.0007 ^a^	657.35 ± 143.02 ^b^	678.33 ± 158.30 ^b^	4.51 ± 0.46 ^b^	0.9446 ± 0.0118 ^a^

Note: a, b, c indicate statistically significant variations in the same column, *p* < 0.05.

## Data Availability

Not applicable.

## Supplementary Materials

The following supporting information can be downloaded at: https://www.mdpi.com/article/10.3390/md20110702/s1, Figure S1: Rarefaction curves and species accumulation box diagram of intestine samples; Figure S2: Venn diagram of operational taxonomic unit (OTU) numbers and rank abundance curve; Figure S3: Heatmap of the species abundance clustering in the top 35 at the genus level; Figure S4: The FTIR spectrum of CS, GT, P-CS-GT (C=N) and CS-GT (C-N); Figure S5: The ^1^H-NMR (a) and solid-state ^13^C-NMR spectrum of CS, OCS, GT, P-CS-GT and CS-GT; Figure S6: The TG curves (a) and DTG curves (b) of P-CS-GT and CS-GT; Table S1: Inhibition rate and IC50 of V. parahaemolyticus dealing with CS, GT and CS-GT; Table S2: The increased multiple of OD260 and K^+^, increased multiple of conductivity dealing with CS, GT and CS-GT at 12 h; Table S3: Composition and relative abundance of gut microbial communities at phylum level; Table S4: Relative intensities of the fitted C1s peak of CS, OCS, GT, P-CS-GT and CS-GT.
