# Supplementary material for: Antibacterial Mechanism of Chitosan–Gentamicin and Its Effect on the Intestinal Flora of Litopenaeus vannamei Infected with Vibrio parahaemolyticus
Source: Mar Drugs. 2022 Nov 9;20(11):702. doi: 10.3390/md20110702 (PMC9697162; doi:10.3390/md20110702)
Supplement: Supplementary file 1 [file marinedrugs-20-00702-s001.zip › marinedrugs-1962288-supplementary.pdf]

Antibacterial mechanism of chitosan-gentamicin and its effect on the intestinal flora of *Litopenaeus vannamei* infected with *Vibrio parahaemolyticus*

Lefan Li [1], Fengyan Liang [1,2,,3]*, Chengpeng Li [1] Tingting Hou[1] and Dong-an Xu[1]

[1] School of Chemistry and Environment Science, Guangdong Ocean University, Zhanjiang 524088, China FLL@gdou.edu.cn (L.L.); lcp0802@126.com (C.L.); htt415@126.com (T.H.)
[2] Life Science &Technology School, LingNan Normal University, Zhanjiang 524048, China
[3] Mangrove Institute, LingNan Normal University, Zhanjiang 524048, China
*Correspondence: yaner204126@163.com; Tel.: +86 18319379880

1.Tables

Table S1. Inhibition rate and IC_{50} of *V. parahaemolyticus* dealt with CS, GT and CS-GT ($\bar{x}$± SD, n=3）

Sample	Inhibition rate(%, 20 μg/mL）	IC_{50}（μg/mL）
CS	12.36±4.10^c	125.62±4.35^a
CS-GT	77.72±5.31^b	18.72±3.17^b
GT	97.38±3.20^a	7.89±5.08^c

Note: a,b,c indicate statistically significant variations in the same column, p <0.01.

Table S2. The increase multiple of OD_{260} and K^+, increase multiple of conductivity dealt with CS, GT and CS-GT at 12 h ($\bar{x}$ ± SD, n=3）

Sample	OD_{260} increase multiple	K^+ increase multiple	Conductivity increase rate（%）
CK	/	/	/
CS	5.80±0.21^c	24.41±4.30^b	8.53±0.05^b
GT	11.56±0.33^a	43.70±4.23^a	17.71±0.05^a
CS-GT	8.62±0.22^b	39.52±5.01^a	15.35±0.04^a

Note: "/" means no data provided; a,b,c indicate statistically significant variations in the same column, p <0.05.

Table S3. Composition and relative abundance of gut microbial communities at phylum level ($\bar{x}$± SD, n=3)

Group	Tenericutes（%）	Proteobacteria（%）	Firmicutes（%）	Bacteroidetes（%）
CK	4.41±0.12^d	34.57±1.53^b	16.33±3.46^b	34.95±1.26^a
Only-infected group	3.32±0.18^d	59.99±1.65^a	21.41±6.96^b	13.65±4.72^c
CS-GT-10	18.68±5.15^c	54.65±1.02^a	5.78±0.32^c	5.86±0.64^d
CS-GT-50	58.25±0.27^b	4.03±0.07^c	11.12±7.72^b	16.01±5.31^c
CS-GT-100	79.05±1.22^a	7.95±0.35^c	3.76±0.35^c	6.78±0.59^d
GT-10	1.68±0.23^d	5.41±0.35^c	37.37±1.09^a	36.52±1.72^a
CS-250	23.75±7.31^c	34.54±1.23^b	34.87±4.41^a	25.26±2.34^b

Note: a,b,c indicate statistically significant variations in the same column, p <0.05

Table S4. Relative intensities of the fitted C1s peak of CS, OCS, GT, P-CS-GT and CS-GT.

Sample	Position (eV)	Relative intensity (%)	Possible element state
CS	285.2	50.73%	C-C/C-H
	286.7	30.16%	C-N
	288.3	13.17%	C-O

	289.2	5.64%	C=O
OCS	285.0	56.27%	C-C/C-H
	286.7	20.53%	C-N
	288.4	10.56%	C-O
	289.2	12.64%	C=O
GT	285.2	50.33%	C-C/C-H
	286.7	36.73%	C-N
	288.4	12.94%	C-O
P-CS-GT	285.0	38.10%	C-C/C-H
	286.7	27.58%	C-N
	287.5	19.16%	C=N
	288.4	10.74%	C-O
	289.3	4.42%	C=O
CS-GT	285.7	42.42%	C-C/C-H
	286.7	29.48%	C-N
	287.5	10.08%	C=N
	288.5	12.24%	C-O
	289.5	5.77%	C=O

2.Figures

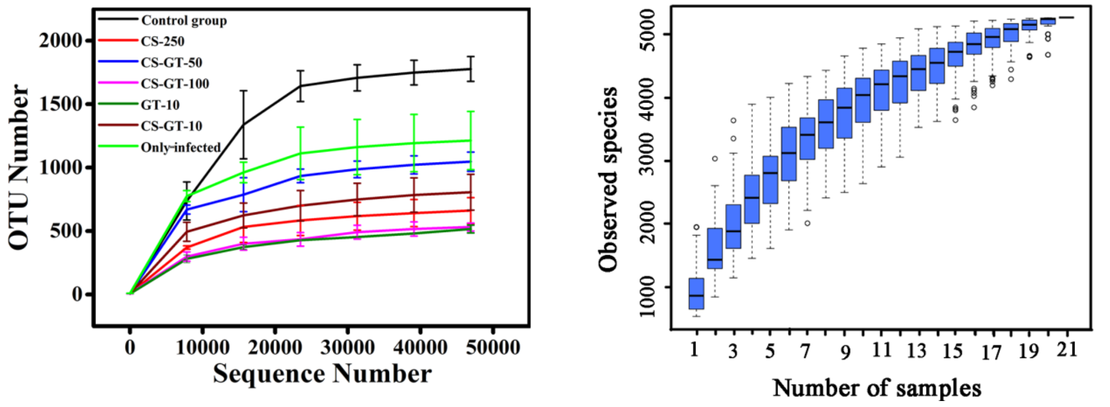

Figure S1. Rarefaction curves and species accumulation box diagram of intestine samples ($\bar{x}$ ± SD, n = 3)

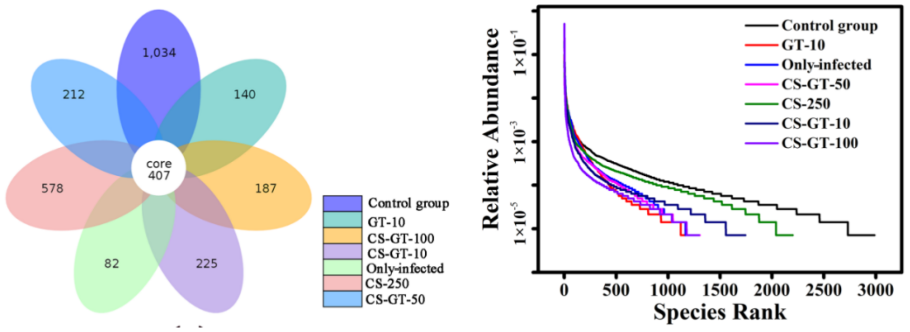

Figure S2. Venn diagram of operational taxonomic unit (OTU) numbers and rank abundance curve ($\bar{x}$ ± SD, n=3)

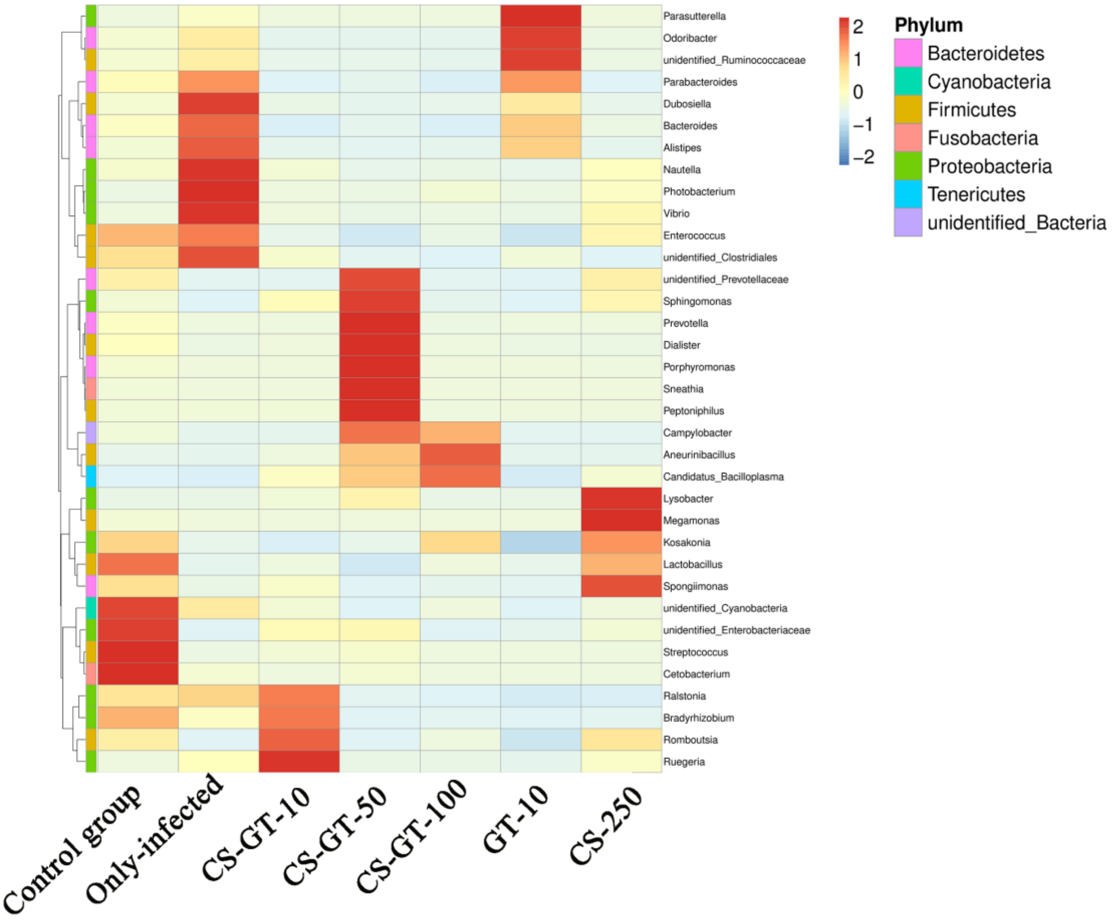

Figure S3. Heatmap of the species abundance clustering in the top 35 at the genus level ($\bar{x}$ ± SD, n=3)

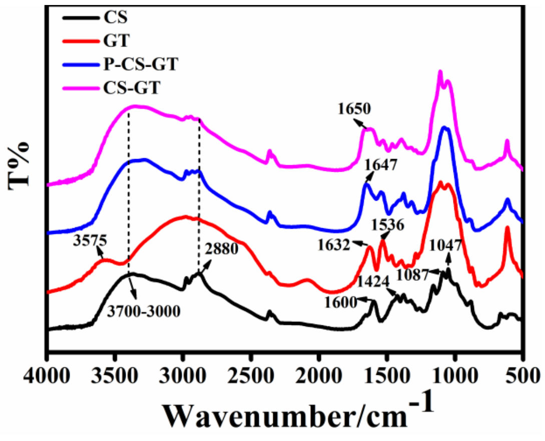

Figure S4. The FTIR spectrum of CS，GT，P-CS-GT (C=N) and CS-GT (C-N).

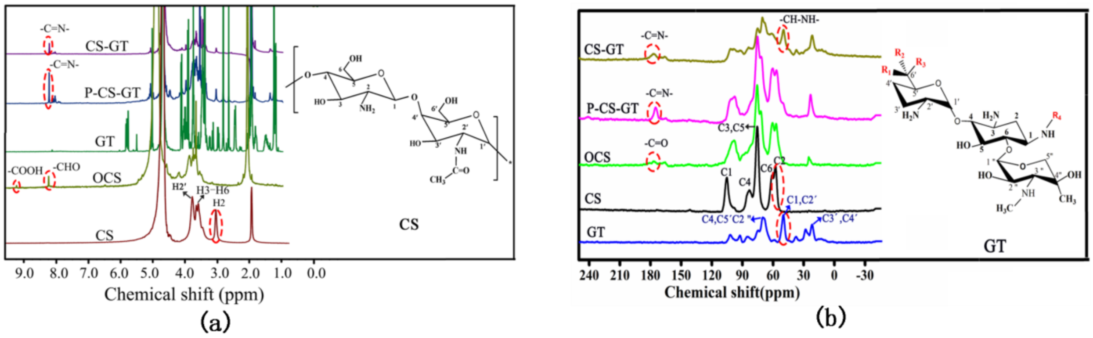

Figure S5. The ^{1}H-NMR (a) and solid-state ^{13} C-NMR spectrum of CS, OCS, GT, P-CS-GT and CS-GT.

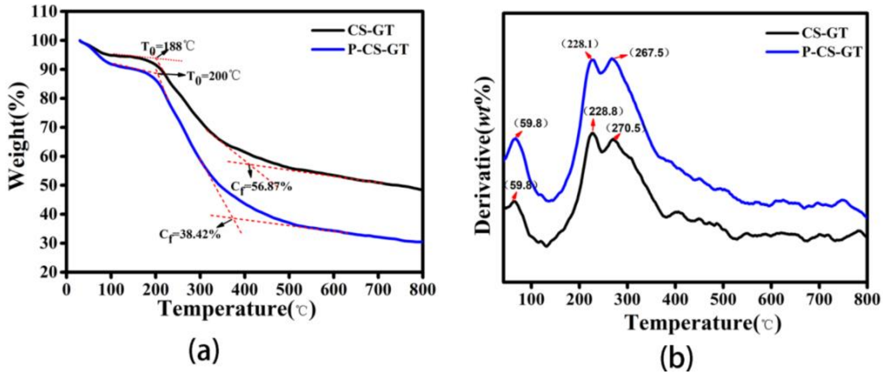

Figure S6. The TG curves (a) and DTG curves (b) of P-CS-GT and CS-GT.
